# A Prototype Antibody-based Biosensor for Measurement of Salivary MMP-8 in Periodontitis using Surface Acoustic Wave Technology

**DOI:** 10.1038/s41598-019-47513-w

**Published:** 2019-07-30

**Authors:** John J. Taylor, Katrin M. Jaedicke, Rachel C. van de Merwe, Susan M. Bissett, Nichola Landsdowne, Kerry M. Whall, Kimberley Pickering, Vivienne Thornton, Victoria Lawson, Hiromi Yatsuda, Takashi Kogai, Deepan Shah, Dale Athey, Philip M. Preshaw

**Affiliations:** 1 Centre for Oral Health Research & Institute of Cellular Medicine, Newcastle University, Newcastle upon Tyne, UK; 2 OJ-Bio, International Centre for Life, Times Square, Newcastle upon Tyne, UK; 3 Orla Protein Technologies, International Centre for Life, Times Square, Newcastle upon Tyne, UK; 4 National University Centre for Oral Health, National University of Singapore, Singapore, Singapore

**Keywords:** Predictive markers, Periodontitis

## Abstract

Periodontitis is an economically important disease which is highly prevalent worldwide. Current diagnostic approaches are time-consuming and require interpretation of multiple aspects of clinical and radiographic assessment. Chair-side monitoring of inflammatory mediators of periodontitis could provide immediate information about disease activity, which can inform patient management. We aimed to develop a novel prototype biosensor to measure salivary matrix metalloproteinase-8 (MMP-8) using specific antibodies and surface acoustic wave (SAW) technology. The analytical performance of the prototype biosensor was compared to standard enzyme-linked immunosorbent assay (ELISA) using unstimulated saliva samples obtained from patients with periodontitis before and after non-surgical treatment (N = 58), patients with gingivitis (N = 54) and periodontally healthy volunteers (N = 65). Receiver operator characteristic (ROC) analysis for distinguishing periodontitis from health revealed an almost identical performance between the sensor and ELISA assays (area under curve values (AUC): ELISA 0.93; SAW 0.89). Furthermore, both analytical approaches yielded readouts which distinguished between heath, gingivitis and periodontitis, correlated identically with clinical measures of periodontal disease and recorded similar post-treatment decreases in salivary MMP-8 in periodontitis. The assay time for our prototype device is 20 minutes. The prototype SAW biosensor is a novel and rapid method of monitoring periodontitis which delivers similar analytical performance to conventional laboratory assays.

## Introduction

Periodontitis is a chronic inflammatory disease that affects the supporting structures of the teeth, leading to tooth mobility and early tooth loss, that has significant impacts on oral function and quality of life. It is an economically important disease that is associated with a number of systemic diseases, and advanced periodontitis has been identified as the 6th most common disease to affect mankind^1^. Early diagnosis of periodontitis enables earlier and more effective intervention and better long term prognosis in addition to facilitating minimally invasive and less time consuming therapies which are more acceptable for patients as well as being economically efficacious.

Current practices for the management of periodontitis are based on interpretation of clinical and radiographic observations rather than objective analysis of the biological factors underlying the disease pathogenesis^2,3^. These procedures are time consuming, require skilled clinicians and are expensive. There is a strong economic prerogative to deliver improved periodontal healthcare in the face of increasing disease prevalence worldwide^4–6^. The increasing appreciation of the clear association of poor periodontal health with common chronic diseases such as cardiovascular disease and diabetes in the wider medical community has highlighted the need for effective diagnostic tests for periodontitis to inform preventative approaches^5^.

Periodontitis tends to proceed in an episodic fashion, with periods of tissue destruction followed by quiescent phases, which may correspond to periods of repair^2,3^. Currently, the tissue breakdown that characterises periodontitis is determined using periodontal probes and radiographs. However, there is a requirement for diagnostic approaches above and beyond traditional approaches based on understanding of the underlying pathogenesis^7^. Such ‘evidence-based knowledge’ is considered critical in preventing clinical mismanagement through the application of inappropriate treatment and failure to correctly characterise active disease^8^.

There is interest in identifying potential biomarkers of periodontitis in oral fluids and investigating their utility in periodontitis diagnosis and longitudinal disease monitoring^9–12^. Saliva is a convenient sampling medium for oral diseases as it is abundant and easily accessible through painless and non-invasive procedures, which do not require sophisticated clinical training to perform^10,13^. The salivary biomarkers, which show most promise in terms of independent replicative studies of disease discrimination and clinical association, have been found to be monocyte-and neutrophil-derived enzymes and, in particular, MMP-8^10,12,14^. Numerous studies confirm that MMP-8 in oral biofluids (and in particular gingival crevicular fluid, GCF) is quantitatively associated with clinical measures of periodontitis both cross-sectionally and longitudinally during treatment^9,10,12^. MMP-8 levels reflect progression of periodontitis and successful treatment and, as such, MMP-8 assays may have positive predictive value for periodontal disease progression^14–17^.

Despite the fact that substantial data endorse the potential of mediators such as MMP-8 as biomarkers of periodontitis, there remains the need to translate this knowledge into so-called ‘high impact diagnostics’ which can significantly uplift clinical decision-making, patient outcomes and oral (and general medical) healthcare economics^18^. Development of point-of-care (POC) devices for the dental clinic are especially attractive as they provide immediate (and objective) information to support patient management and are potentially transferrable to other environments such as the home, the care-home, and ‘in the field’ in areas where clinical facilities are not available.

The fundamental requirements for diagnostic tests for periodontitis have been defined^19^ but, clearly, new diagnostic approaches will need to be fit for clinical purpose in terms of analytical performance before ergonomics and economic factors are considered. Several analytical platforms to measure MMP-8 have been proposed to have utility as POC devices for periodontitis^12,17^. Some approaches rely on the collection of GCF^12^, a procedure which requires considerable clinical expertise and is fraught with technical challenges in terms of quantification of soluble mediators^20^. It is well documented that saliva collection is more convenient and acceptable to the patient and, furthermore, salivary mediators are reflective of biological processes in the whole mouth rather than at individual periodontal sites (as with GCF)^10^.

The only currently marketed POC device for salivary molecular biomarker analysis is the Periosafe^®^ test which combines lateral flow technology with ELISA detection chemistry to detect active MMP-8 (aMMP-8) in mouth rinse samples^17,21^. Other prototype POC tests which measure neutrophil elastase (MMP-9) in GCF and salivary C-reactive protein have been described but information on their clinical utility is, thus far, limited^22^.

Also, tests based on the detection of neutrophil aspartate aminotransferases in GCF have been described, although these tests are compromised by complex sample processing steps and unsatisfactory correlation of readouts with clinical periodontal parameters^23,24^. Other platforms for multiplex analysis of soluble mediators including cytokines and MMPs have been outlined in preliminary reports which remain to be followed by substantial analytical or clinical performance data^25–27^.

The prototype biosensor we describe herein captures MMP-8 using specific antibodies coated on a small biochip, and quantifies salivary MMP-8 via microelectromechanical piezoelectric SAW technology^28,29^. The biosensor provides a quantitative readout proportional to levels of salivary MMP-8. Furthermore, the biosensor assay exhibits an analytical performance comparable to that of ‘gold standard’ ELISA assays for MMP-8, shows significant correlation with conventional clinical parameters of periodontitis, clearly distinguishes between periodontitis and gingivitis, and tracks MMP-8 changes after clinical treatment.

## Results

### Analytical characteristics of the biosensor assay of MMP-8

**Figure 1:**
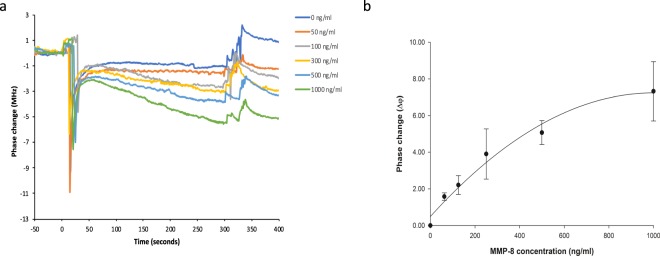
Response of the biosensor to hrMMP-8. Biosensor phase change response to increasing concentrations of hrMMP-8 (0–1000 ng/ml). Overlaid plots of raw data from a single experiment showing phase shifts recorded as a function of time after the addition of the detection antibody at t = 0 (**a**). Graph illustrating the relationship of relative phase change between samples containing MMP-8 and controls (Δφ) (**b**). Phase change measurements were made after final buffer wash (see Methods). Data are derived from 3 independent experiments each comprising parallel duplicate measurements on separate microchips and are presented as means ± SD (n = 6).

The dose-response performance for analysis of human recombinant MMP-8 (hrMMP-8, Biotechne) in buffer is illustrated in Fig. 1. The effective range for this assay is 0–1000 ng MMP-8/ml with an estimated limit of detection of 62.5 ng/ml. Although the limit of detection is not the same as ELISA^30^, the biosensor performed as well as the ELISA in the clinically relevant range (Fig. 1a). To assess the reproducibility of the SAW biosensor assay, 3 saliva samples with range of different MMP-8 levels were analysed by the biosensor in quadruplicate in one assay (intra-assay variation) on the same biochip and one saliva sample tested in quadruplicate on 3 consecutive days (inter-assay variation) on separate biochips; these experiments provided both intra- and inter-assay variation of 13.2% which is slightly higher than similar data for the Quantikine ELISAs (8% for both inter- and intra-assay)^30^. In a later iteration of the analysis protocol, direct binding of capture antibody anti-MMP-8 to sensor chip (omitting the anti-Ig antibody) reduced assay time and improved reproducibility with no effect on performance. Thus, biochips were used in the following protocol: incubation with 20 μl TBS-T for 1 minute, 20 μl of sample containing MMP-8 (standard or saliva) was then added for 5 minutes followed by 5 x TBS-T for 2 minutes, secondary anti-MMP8 antibody for 5 minutes followed by a 1 minute wash with 5 x TBS-T. This protocol yielded similar standard curves (not shown) but with reduced assay time (15 minutes) and improved reproducibility (8.6% and 9.1% intra-assay and inter-assay variability respectively).

### Comparative analysis of salivary MMP-8 by ELISA and biosensor methods

**Figure 2:**
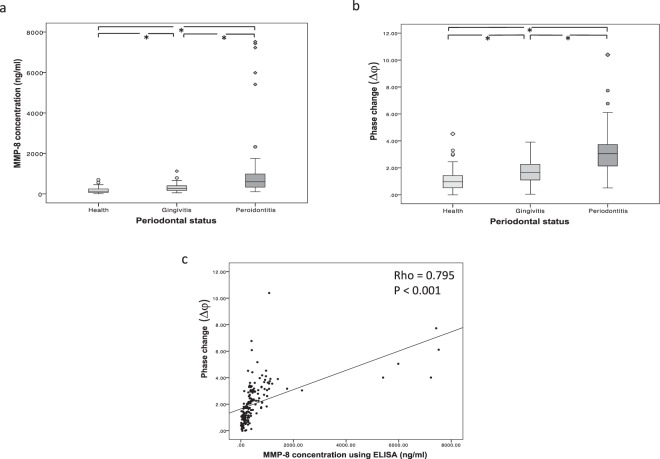
Analytical performance of the biosensor for the assay of salivary MMP-8 in periodontal disease. Analysis of salivary MMP-8 in healthy volunteers (N = 56), patients with gingivitis (N = 47) and patients with periodontitis (n = 65) using ELISA (**a**) and the biosensor assay (**b**). Data are means of duplicate measurements and are presented as box and whisker plots: boxes represent median (line) and interquartile ranges and whiskers the minimum and maximum range. Data was analysed using Kruskal-Wallis one-way ANOVA with Mann-Whitney U post hoc test, *P < 0.001. Correlation of salivary MMP-8 analysis of clinical samples (n = 168) using ELISA and biosensor assays (**c**). Data were analysed using Spearman’s Rank correlation, Rho = 0.795, P < 0.001.

**Figure 3:**
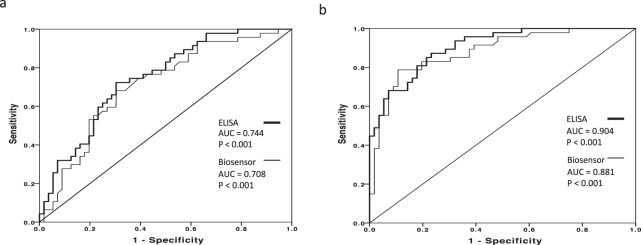
Diagnostic performance of salivary MMP-8 analysis using the biosensor and ELISA in periodontal disease. Comparative receiver operator characteristic (ROC) plots for salivary MMP-8 measurements using ELISA and the biosensor assay (BS) assays. Sensitivity and specificity of gingivitis diagnosis (vs health) (**a**) and periodontitis diagnosis (vs health) (**b**) are presented and AUC data and P values included.

Analysis using both the ELISA and the new MMP8 biosensor indicated that the levels of salivary MMP-8 were significantly different (P < 0.001) between healthy volunteers, patients with untreated gingivitis and patients with untreated periodontitis (Fig. 2a,b). Furthermore, there was a statistically significant correlation between the analytical readouts for both assays (Fig. 2c). The broad range of values for MMP-8 levels reflects the unbiased inclusion of values from the analysis of samples from all 3 clinical groups. ROC analysis revealed that both ELISA and sensor analysis for salivary MMP-8 provided similar sensitivity and specificity in terms of distinguishing MMP-8 levels in healthy individuals from those in both gingivitis patients (Fig. 3a) and periodontitis patients (Fig. 3b).

### Analysis of MMP-8 in relation to disease severity and treatment

**Figure 4:**
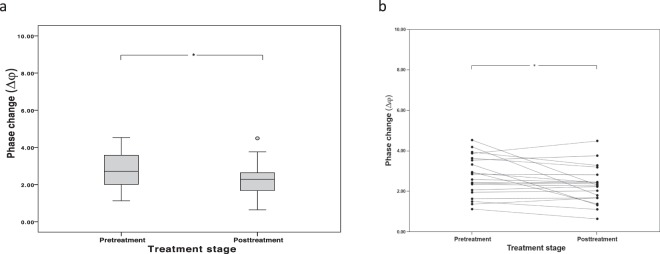
Comparative biosensor analysis of salivary MMP-8 before and after treatment for periodontitis. Analysis of salivary MMP-8 in a sub-group of patients (n = 20) before and after 6 months after non-surgical treatment for periodontitis. (**a**) Data presented as ‘box and whisker plots’: boxes represent median (line) and interquartile ranges and whiskers the minimum and maximum range. Data were analysed using a paired samples t-test *P = 0.026. (**b**) Figure showing the changes in levels on MMP-8 before and after treatment in saliva samples from individual patients.

Salivary MMP-8 measurements using both analytical methods correlated similarly with clinical measures of periodontitis taken at the beginning of study (Table 1). Thus, there were significant correlations between salivary MMP-8 levels as measured by the biosensor and ELISA methods and bleeding on probing (BOP) (P = 0.022 and P = 0.005 respectively), periodontal probing pocket depth (PPD) (P = 0.001 and P = 0.002 respectively), periodontal inflamed surface area (PISA) (both P = 0.003), and periodontal epithelium surface area (PESA) (P = 0.001 and P = 0.004 respectively), but not mean gingival index (MGI) (P = 0.0228 and P = 0.272 respectively), or clinical attachment loss (CAL) (P = 0.734 and P = 0.092 respectively), (Table 1). The strength of correlations between the clinical measurements and MMP-8 were similar using both the ELISA and the biosensor assays (Table 1). We also tested the ability of the biosensor assay to measure changes in MMP-8 6 months after the commencement of non-surgical treatment for periodontitis. In agreement with the literature, ELISA measurements of MMP-8 before and after treatment in 62 paired pre-treatment and post-treatment samples demonstrated a significant reduction in salivary MMP-8 levels (P < 0.001, Supplemental Fig. 1). We then assayed MMP-8 in saliva taken from a sub-group (n = 20) of periodontitis patients using the biosensor assay and recorded a similarly significant reduction in posttreatment MMP-8 levels in the group as a whole (P = 0.026, Fig. 4a). It is worthy of note that MMP-8 levels did not decline post-treatment in a minority of patients in this cohort (Fig. 4b).

**Table 1:** Correlation of salivary MMP-8 assays with clinical parameters of periodontitis as assessed in untreated periodontitis patients (N = 65).

Clinical periodontal measurement	Assay for MMP-8			
	Biosensor phase change		ELISA	
	Spearman’s rho	P	Spearman’s rho	P
Bleeding on probing (BOP)	0.287	0.022	0.347	0.005
Mean gingival index (MGI)	0.153	0.228	0.139	0.272
Probing pocket depth (PPD)	0.403	0.001	0.382	0.002
Clinical attachment loss (CAL)	0.043	0.734	0.212	0.092
Periodontal inflamed surface area (PISA)	0.367	0.003	0.369	0.003
Periodontal epithelial surface area (PESA)	0.391	0.001	0.359	0.004

### Prototype interleukin-1β (IL-1β) sensor

In preliminary experiments we have extended our experimental approach to develop a biosensor to measure salivary IL-1**β** which is similarly sensitive and reproducible (Supplemental Fig. 2a). Also, the prototype IL-1**β** sensor provides measurements which correlate with those derived from ELISA measurements (Supplemental Fig. 2b).

## Discussion

The aim of the present study was to develop a novel prototype biosensor to measure salivary MMP-8 using specific antibodies and SAW technology. We selected MMP-8 because of its known role in the pathogenesis of periodontal disease and a substantial literature supporting its role as a biomarker of gingivitis and periodontitis. Using ELISA we confirmed the utility of MMP-8 measurements using the biosensor in distinguishing periodontal health from gingivitis and periodontitis, the quantitative association of salivary MMP-8 levels with severity of periodontitis and the association of reduced levels with clinical improvement after non-surgical treatment for this disorder. These data are comparable to previously reported data for the analysis of MMP-8 in saliva collected by passive drooling^15,31–33^. We successfully developed a protocol for the construction and operation of a prototype biosensor to measure total MMP-8 in human saliva. The analytical performance of the biosensor in terms of sensitivity and reproducibility of MMP-8 analysis compared favourably to that of the commercial ELISA kit and the data in terms of clinical association of measured MMP-8 was almost identical. We did identify some 7 periodontitis patients with MMP-8 levels >2000 pg/ml which are outwith the range of data we recorded for other patients but within the range recorded in a meta analysis of other studies of salivary MMP-8 levels^34^. The wide range of inter-subject variability in salivary MMP-8 levels may be clinically relevant and may be useful in developing novel paradigms for biomarker analysis in periodontitis which may extend their clinical utility^12,35^.

The biosensor has the advantage of real-time measurements, and modification of protocols for chip preparation and washing allowed the reduction in time of analysis to approximately 15 minutes. Also, unlike many lateral flow-type sensors, the sensor system has a scalable readout rather than relying on the appearance of colours or bands. Clearly, as previously outlined^28^, there are aspects of the biosensor that currently limit the utility of this device. Activation of biochip is a procedure which is carried out by manual steps which may influence chip-to-chip reproducibility; this can be improved by using automated inkjet printing which also facilitates mass production^28,29^. There are multiple assay steps which require manual pipetting; in our development work, we have addressed this by omitting an antibody binding step which has reduced assay time and developments are already underway to incorporate all components into an integrated reaction cassette, during mass manufacture, which allows for simple one-step sample addition and subsequent read-out. The cassette also incorporates developments in microfluidics that facilitate sample mixing without multiple pipetting steps^36^. Also, the laboratory prototype biosensor described herein currently employs simultaneous duplicate measurements using 2 biochips; use of a reference biochip^28^ or even a dual channel biochip with an *in situ* reference^29^ reduces the effects of both inter-chip variation and non-specific effects such as temperature and sample viscosity variation. There are opportunities to develop a more streamlined sample collection regimen, for example, collecting a mouth rinse rather than saliva by passive drooling and filtration of oral fluid through syringes rather than centrifugation as has been employed with other POC devices^21^.

There are several other point-of-care devices designed to aid periodontal disease management which have been reported in the literature. The Periosafe^®^ test combines lateral flow technology with ELISA detection chemistry to detect active MMP8 (aMMP-8) in mouth rinse samples^17,21,37,38^. The ‘readout’ comprises the appearance of lines of varying intensity: a light line indicates low ‘risk’ and a dark line high ‘risk’^37^. The test has a limit of detection of 20–25 ng/ml aMMP-8. The results of analysis using this kit correlated with poor oral hygiene, periodontitis and BOP^38^ and has an overall sensitivity of 76–90% and specificity of 96% in the diagnosis of periodontitis although there are no data for gingivitis^21,37–39^. The Periosafe^®^ test also gives readouts which correlate with quantitative reductions in MMP-8 levels post-treatment^17^. The Periosafe^®^ test employs a proprietary antibody to aMMP-8 and therefore only assays the enzymatically active fraction of total MMP-8. The biosensor, like the ELISA kit used in this study (Quantikine, R&D Systems), employs antibodies to total MMP-8 (which includes the inactive pro-MMP-8 fraction). However, the analytical and clinical performance characteristics of the Periosafe^®^ test are similar to the biosensor assay platform described herein. Other research recording the utility of salivary MMP-8 assays in diagnosis of periodontitis has likewise employed assays using antibodies to total MMP-8 in ELISA assays^15,33^.

It is possible that assay of oral MMP-8 (in the form of GCF, saliva or oral rinse) has positive predictive value for clinical outcome in periodontitis. The ELISA and biosensor assays both recorded a significant reduction in salivary MMP-8 after periodontitis treatment in the group as a whole. Although, in agreement with other studies ^14,15^, we noted some inter-individual variability in the longitudinal changes in MMP-8 levels post-treatment^14,15^. A reduction in levels of MMP-8 in oral fluids (principally GCF) is associated with successful treatment for periodontitis^12^. A recent study using both the Periosafe^®^ test and an immunoassay revealed a remarkably consistent reduction of aMMP-8 as measured in oral rinse samples 6 weeks post-treatment in 10 patients^17^. Clearly, more substantial, longitudinal clinical studies of salivary biomarkers in periodontitis are warranted before their clinical utility, and that of POC devices including the prototype sensor described herein, which analyse them, can be determined^17^.

It is recognised that other salivary biomarkers (e.g. MMP-9, IL-1**β**) may be clinically useful either when measured in isolation or in combination with other candidate biomarkers such as MMP-8^12,15,31–33,40^. The prototype biosensor system we have developed employs a simple capture and detection dual antibody system and is adaptable to the assays of other analytes. Indeed, we have developed similar biosensor assays for IL-1**β** (Supplementary data). This opens up the possibility of developing sensors based on simultaneous multiplex analysis for different analytes. We also note that assay of biomarkers in oral fluids has potential application to the clinical monitoring of other oral inflammatory conditions such as peri-implantitis^21^.

The current research is based on biochips in a laboratory prototype sensor; for commercial development the biochip will be packaged in a disposable cassette which can send data to a smartphone via Bluetooth; the potential physical and functional characteristics of such a portable hand-held device have been outlined in detail elsewhere^28,29^. In line with other POC devices, the speed of the test will support patients, carers and health-care professionals by widening access to objective clinical measurements outside the clinical setting^41^. This will be particularly important given the high prevalence of periodontitis and the increasing recognition of the clinical relationship between periodontitis and a number of common chronic conditions including, in particular, diabetes and cardiovascular disease.

## Materials and Methods

### Prototype sensor for MMP-8

The prototype sensor comprises a disposable SAW biochip functionalised with specific antibodies which delivers an analogue signal to a control box upon analyte detection. The control box converts the signal to a digital format which is received by a laptop PC with dedicated software to process the signal. The physical structure and microelectronic architecture of the biochips used in the sensor have been described previously^28,42^. Briefly, the biochip comprises interdigitating input and output gold electrodes linked by a gold film coated sensing area built on a plane piezoelectric quartz crystal. This structure facilitates excitation of a shear horizontal SAW of defined wavelength and frequency. The biosensor has a protective glass covering and epoxy walls constructed as previously described^42^.

Capture antibodies on the surface of the gold film and this biochip thereby become sensitive to reactions (e.g. antigen binding) by means of a SAW velocity and /or amplitude changes due to surface condition changes^28,42^. Thus, upon application of a biological fluid containing antigen (e.g. MMP-8) the mass and/or viscosity perturbation caused by antigen/antibody binding is detected by the difference in wave phases between the input and output electrodes (i.e. phase change, Δφ).

### Biochip functionalization

**Figure 5:**
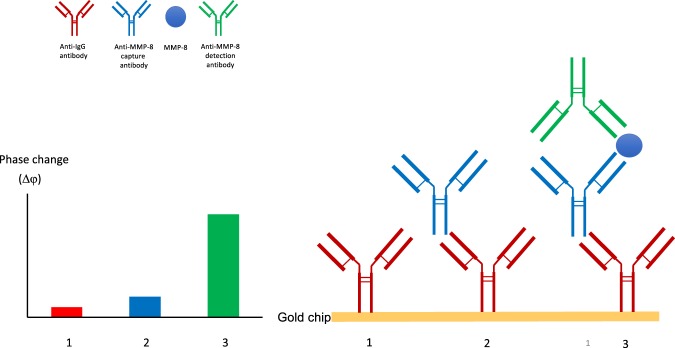
Molecular interactions at the surface of the biosensor biochip. Schematic illustrating the functionalisation of the gold-coated biochip with firstly anti-IgG antibodies (red), and secondly with anti-MMP-8 capture antibodies (blue), followed by detection by successive addition of sample (containing MMP-8) and anti-MMP-8 detection antibody (green). The binding of the anti-MMP-8 detection antibody to MMP-8 bound to the immobilised complex results in a perturbation of mass at the biochip surface sufficient to generate a phase change (Δφ) in the surface acoustic wave detected by the biosensor control box. In later experiments, the anti-MMP-8 capture antibody was bound directly to the biochip, omitting the anti-IgG antibody step. A full description of the prototype biosensor structure, microelectronics and detection system has been recently published^28^.

The architecture of functionalised biochip and the antibody/analyte interactions are illustrated in Fig. 5. Anti-Ig antibodies (polyclonal anti-mouse F_c_, Jackson ImmunoResearch Europe Ltd, Ely, UK) were used to bind an analyte-specific primary antibody. This primary antibody subsequently captures the analyte, the presence of which is detected when the secondary antibody binds the immobilised immune complex. The antibodies used were: human MMP-8 capture antibody (mouse monoclonal, MAB908, Biotechne, Abingdon, UK), human MMP-8 detection antibody (goat polyclonal, AF908, Biotechne). The functionalisation protocol for the biochips was as follows: the SAW biochips were cleaned in a 2% (v/v) solution of Hellmanex III (Hellma Analytics, Southend, UK) followed by copious washings with deionized water. Biochips were activated by incubation with a 4 mg/ml solution of dithiobis [succinidyl propionate] (DSP, Thermo Fisher Scientific, Loughbourgh, UK) in DMSO (Thermo Fisher Scientific) followed by washes with DMSO and PBS. Subsequently the biochips were functionalised by incubation of 100 μg/ml anti-Ig antibody in PBS followed by a PBS wash. Unreacted DSP was inactivated by successive washing with TBS-T buffer (Tris-buffered saline at pH7.6 with 0.05% v/v Tween 20). The surface was incubated with 2% (w/v) bovine serum albumin (BSA, Sigma Aldrich, Gillingham, UK) to block non-specific binding.

### Testing protocol

The biochips were placed in a receptacle on a laboratory prototype biosensor device connected to a control box for data logging and transmission to laptop for processing. This can simultaneously read the phase changes from two biochips independently. The output data were, therefore, the mean of 2 simultaneous measurements of the same sample. A stable buffer baseline was established with 20 μl TBS-T. The testing protocol comprised successive incubations with 20 μl of reagents as follows: TBS-T for I minute, 100 mM HCl for 2 minutes, wash with 5 volumes of TBS-T for 2 minutes, anti-MMP-8 capture antibody (30 μg/ml) for 5 minutes, wash 5 x with TBS-T for 2 minutes, sample for 5 minutes, TBS-T wash 5x for 2 minutes, anti-MMP-8 detection antibody (30 μg/ml) for 5 minutes, wash 5x with TBS-T to get a final reading. The surface was regenerated with 100 mM HCl and finally washed 5x with TBS-T. Note that the protocol was optimised during the study (see Results section).

### ELISA

Salivary MMP-8 was measured using ELISA (Quantikine, Biotechne) according to the manufacturer’s instructions. Samples were analysed in duplicate, absorbance read at 450 nm on a FL600 Microplate Reader (Biotek, Swindon, UK) and MMP-8 concentrations calculated from standards by means of a 4-parameter logistic curve fit using the proprietary software (KC4 KinetCalc, Biotek).

### Clinical samples

Three subject groups were recruited as part of this study: periodontally healthy volunteers (N = 56), patients with gingivitis (N = 47), and patients with chronic periodontitis (N = 65). All participants were adult males or females aged between 18 and 65 with a minimum of 20 natural teeth (excluding 3^rd^ molars) and were non-smokers. The diagnostic criteria were as follows: healthy participants had PPD of ≤ 3 mm in all sites, no sites with interproximal attachment loss, MGI scores of ≥ 2.0 in ≤ 10% of sites and %BOP scores of ≤ 10%; gingivitis patients had MGI of ≥ 3.0 in ≥ 30% of sites, no sites with interproximal attachment loss, PPD > 4 mm and %BOP scores of ≥ 10%; periodontitis patients had interproximal PPD of ≥ 5 mm at ≥ 8 teeth and % BOP scores of ≥ 30%. All subjects provided written informed consent, the study was conducted at the Dental Clinical Research Facility of Newcastle Dental Hospital (part of the Newcastle upon Tyne Hospitals NHS Foundation Trust) and the study was fully approved by the National Research Ethics Service North East Newcastle and North Tyneside 1 committee (Ref: 12/NE/0396). All methods were performed in accordance with relevant guidelines and regulations. Subjects with evidence of infectious or systemic disease, currently undergoing treatment with antibiotics or immunosuppressants or those who had smoked within the last 2 years were excluded from the study. Full mouth periodontal clinical indices were recorded, and saliva samples were collected at month 0 (all subjects) as well as 3 months post-treatment (gingivitis and periodontitis patients), and 6 months (periodontitis patients). Periodontal clinical indices were recorded at 6 sites per tooth and included GI, PPD, gingival recession, CAL and % BOP. PISA and PESA were calculated as previously published^43^. Unstimulated saliva samples (3–5 ml) were collected by expectoration into a plastic centrifuge tube, placed on ice immediately after collection and centrifuged for 15 minutes at 1500 g and at 4 °C. Aliquots were frozen in liquid nitrogen and stored at −80 °C until analysis. PISA and PESA were calculated as previously published^43^. Unstimulated saliva samples (3–5 ml) were collected by expectoration into a plastic centrifuge tube, placed on ice immediately after collection and centrifuged for 15 minutes at 1500 g and at 4 °C. Aliquots were frozen in liquid nitrogen and stored at −80 ^o^C until analysis.

### Statistical analysis

Differences in MMP-8 levels between patient groups were determined by Kruskal-Wallis one-way ANOVA with Mann-Whitney U *post hoc* tests with adjustment of the critical value of P as appropriate. Correlations between MMP-8 levels measured by ELISA and the sensor as well as with clinical parameters were carried out using Spearman’s Rank correlation. Specificity and sensitivity of analyte measurements in the detection of periodontal diseases were determined by ROC analysis. Differences in phase shift results between pre-treatment and post-treatment periodontitis saliva samples were determined using paired samples t-tests. All statistical analyses were carried out using SPSS version 24.0.0 (IBM, Portsmouth, UK). *P* values of <0.05 were considered statistically significant.

## Supplementary information

Supplementary material

## Data Availability

The database used in this study includes clinical and laboratory data collected prospectively from consented volunteers and patients. This database contains protected health information and, in accordance with the ethical approval, has not been made publicly available.
