# Supplementary material for: A Prototype Antibody-based Biosensor for Measurement of Salivary MMP-8 in Periodontitis using Surface Acoustic Wave Technology
Source: Sci Rep. 2019 Jul 30;9:11034. doi: 10.1038/s41598-019-47513-w (PMC6667706; doi:10.1038/s41598-019-47513-w)
Supplement: Supplementary file 1 — Supplementary material [file 41598_2019_47513_MOESM1_ESM.docx]

**SUPPLEMENTAL MATERIAL**

**A Prototype Antibody-based Biosensor for Measurement of Salivary MMP-8 in Periodontitis using Surface Acoustic Wave Technology**

John J. Taylor^1*^, Katrin M. Jaedicke^1^, Rachel C. van de Merwe^1^, Susan M. Bissett^1^, Nichola Landsdowne^1^, Kerry M. Whall ^1^, Kimberley Pickering^1^, Vivienne Thornton^2^, Victoria Lawson^2^, Hiromi Yatsuda^2^, Takashi Kogai^2^, Deepan Shah^3^, Dale Athey^2,3^, Philip M. Preshaw^1,4^

^1^Centre for Oral Health Research & Institute of Cellular Medicine, Newcastle University, Newcastle upon Tyne, UK; ^2^OJ-Bio, International Centre for Life, Times Square, Newcastle upon Tyne, UK; ^3^Orla Protein Technologies, International Centre for Life, Times Square, Newcastle upon Tyne, UK; ^4^National University Centre for Oral Health, National University of Singapore.

*Corresponding author: john.taylor@ncl.ac.uk.

**Supplemental figure 1**

COMPARATIVE ELISA ANALYSIS OF SALIVARY MMP-8 BEFORE AND AFTER TREATMENT FOR PERIODONTITIS.

Fig.1 ELISA analysis of salivary MMP-8 in a sub-group of patients (n=62) before and 6 months after non-surgical treatment for periodontitis. Data are means of duplicate measurements and are presented as box and whisker plots: boxes represent median (line) and interquartile ranges and whiskers the minimum and maximum range. Data were analysed using a paired samples t-test after log transformation to achieve normal distribution *P<0.001.

**Supplemental figure 2**

DEVELOPMENT OF A PROTOTYPE BIOSENSOR MEASURING SALIVARY IL-1β

Fig.2. Dose-response and reproducibility a biosensor for IL-1β. Biochips were functionalised and prepared for testing as described in the Methods. The testing protocol comprised successive incubations with 20 μl TBS-T for I minute, 20 μl of anti-IL-1β capture antibody (30 μg/ml, mouse monoclonal MAB 601, Biotechne) for 5 minutes followed by a 5xTBS-T for 2 minutes. Sample containing IL-1β (standard or saliva) was then added (20 μl) for 5 minutes followed by 5xTBS-T for 2 minutes. 20 μl of anti-IL-1β detection antibody was then added (30 μg/ml biotinylated goat polyclonal BAF 201, Biotechne) for 5 minutes followed by 5xTBS-T for 2 minutes. 20 μl of neutravidin-gold conjugate (ThermoFisher Scientific) was then added for 2 minutes followed by 5xTBS-T for 2 minutes.

Biosensor phase change (Δϕ) response to increasing concentrations of hrIL-1β (0-1000 ng/ml) is illustrated in Fig.2a. Data are derived from a 2 experiments each comprising parallel duplicate measurements on separate microchips and are presented as means ± SD (n=4). To assess the reproducibility of the biosensor assay, 3 saliva samples with range of different IL-1β levels were analysed by the biosensor in quadruplicate in one assay (intra-assay variation) and one saliva sample was tested in quadruplicate on 3 consecutive days (inter assay variation); these experiments provided an intra-assay variation of 12.9% and an inter-assay variation of 7.8% comparable in performance to both the MMP-8 biosensor assay (see Results) and the IL-1β ELISAs *(30)*. A Spearman's rank-order correlation was run to determine the relationship between salivary IL-1β (n=24) as assayed by ELISA (Quantikine, Biotechne) and the biosensor (Fig.2b). There was a positive correlation between these two assays which was statistically significant (Rho=0.485, P=0.016)
